# Suprachoroidal triamcinolone acetonide versus rescue therapies for the treatment of uveitic macular oedema: A post hoc analysis of PEACHTREE

**DOI:** 10.1111/ceo.14024

**Published:** 2021-12-27

**Authors:** Michael A. Singer, Pauline Merrill, Steven Yeh, Colette Hall, Barry Kapik, Thomas A. Ciulla

**Affiliations:** ^1^ University of Texas Health Science Center San Antonio Texas USA; ^2^ Medical Center Ophathlmlogy Associates San Antonio Texas USA; ^3^ Illinois Retina Associates Chicago Illinois USA; ^4^ Rush University Medical Center Chicago Illinois USA; ^5^ University of Nebraska Medical Center Omaha Nebraska USA; ^6^ Clearside Biomedical Alpharetta Georgia USA

**Keywords:** macular oedema, retina, steroids, suprachoroidal, uveitis

## Abstract

**Background:**

This post hoc analysis compared the efficacy and safety of suprachoroidally administered triamcinolone acetonide (CLS‐TA) to other commonly available treatments for non‐infectious uveitis.

**Methods:**

Results from the PEACHTREE study were compared between subjects randomised to CLS‐TA not requiring rescue therapy and those subjects randomised to control, who subsequently required rescue therapy. Endpoints included best corrected visual acuity (BCVA), central subfield thickness (CST), treatment emergent adverse events and intraocular pressure (IOP) related safety findings.

**Results:**

In this analysis, there were 83 unrescued CLS‐TA subjects and 46 rescued control subjects. At Week 24, 51.9% of the unrescued CLS‐TA subjects gained ≥15 letters in BCVA, compared to 37.0% of the rescued control subjects (*p* = 0.115). Unrescued CLS‐TA subjects showed a mean gain of 15.7 versus 10.9 letters in rescued control subjects (*p* = 0.080). A significantly greater mean reduction in CST was observed for unrescued CLS‐TA subjects versus rescued control subjects (174.0 and 148.5 μm; *p* = 0.040). Of unrescued CLS‐TA subjects, 4.9% experienced IOP elevations ≥30 mm Hg at any visit versus 10.9% of rescued control subjects. Further, use of IOP‐lowering medications appeared lower in unrescued CLS‐TA subjects versus rescued control subjects (7.2% vs. 13.0%). There were no IOP‐lowering surgical interventions in either group.

**Conclusion:**

CLS‐TA subjects experienced significantly greater reduction in CST and tended towards greater improvement in BCVA, compared with rescued control subjects. Suprachoroidally administered CLS‐TA showed a lower incidence of IOP‐related safety findings.

## 1 INTRODUCTION

Uveitis is a common cause of a legal blindness, accounting for 10%–15% of cases in the developed world.^1^, ^2^, ^3^ Approximately 30% of uveitis patients develop macular oedema (ME).^4^ Uveitic ME and inflammation are often treated with intravitreal (IVT) corticosteroids, but anterior segment exposure leads to ocular hypertension in 20%–60% cases, as well as exacerbation of glaucoma and cataract.^5^, ^6^, ^7^, ^8^, ^9^ In pre‐clinical studies, investigational suprachoroidal administration of triamcinolone acetonide (CLS‐TA) targets affected posterior tissues, while limiting corticosteroid exposure to the anterior segment, potentially decreasing the incidence of these adverse events (AEs).^10^, ^11^, ^13^

Suprachoroidal injection of CLS‐TA was assessed in the phase 3 PEACHTREE study (NCT02595398) in subjects with non‐infectious uveitis (NIU) complicated by ME.^12^ PEACHTREE was a masked, randomised trial with 160 subjects randomised in a 3:2 ratio to 4 mg CLS‐TA or control (sham procedure) with two administrations 12 weeks apart. In the CLS‐TA arm, 46.9% of subjects gained ≥15 Early Treatment Diabetic Retinopathy Study (ETDRS) letters in best corrected visual acuity (BCVA) versus 16% in the control arm (*p* < 0.001) at Week 24, meeting the primary endpoint. Compared to baseline, ME, as measured by central subfield thickness (CST), reduced by a mean of 153 µm versus 18 μm (*p* < 0.001) for the CLS‐TA and control arms, respectively. All subjects were allowed to receive rescue therapy based on a set of pre‐defined criteria or at the investigators' discretion. The control arm received rescue therapy more frequently (71.9%) compared to the CLS‐TA arm (13.5%; *p* < 0.001), with IVT and periocular corticosteroids being the most commonly prescribed rescue therapies. AEs of elevated intraocular pressure (IOP), not temporally related to the injection procedure, occurred in 11.5% of CLS‐TA arm and 15.6% of the control arm, likely reflecting the more frequent IVT and periocular corticosteroid rescue in the control arm. Cataract AE rates were comparable (7.3% and 6.3%) in the CLS‐TA arm versus the control arm.

Suprachoroidal administration, with anatomically precise drug delivery, has the potential to yield durable safety and efficacy benefits over current therapies.^13^ However, as with other NIU corticosteroid therapies that have utilised sham‐controlled trial designs as a basis for authorization, PEACHTREE did not compare suprachoroidal administration of CLS‐TA to other commonly available treatments. Consequently, the primary objective of this post hoc analysis was to evaluate PEACHTREE safety and efficacy results from CLS‐TA subjects who were unrescued to those from rescued control subjects.

## 2 METHODS

### 2.1 Study participants/design

The PEACHTREE full study design and main findings have been reported elsewhere.^12^ PEACHTREE was conducted in accordance with the ethical principles of Good Clinical Practice, according to the ICH Harmonised Tripartite Guideline and the Declaration of Helsinki; institutional review board committee approval was obtained. All study patients provided informed consent. To be eligible in the original study, subjects had to have a diagnosis of NIU of any aetiology and have ME secondary to uveitis, with a retinal thickness of ≥300 μm in the central subfield as measured by spectral‐domain optical coherence tomography. Subjects were required to have a BCVA score of ≥5 ETDRS letters (20/800 Snellen equivalent) and ≤ 70 letters (20/40 Snellen equivalent) in the study eye. Subjects meeting eligibility were randomised to 4 mg of CLS‐TA administered via suprachoroidal injection or to control (sham procedure) at Day 0 and Week 12. Ocular and safety assessments were performed at each monthly visit as previously described.^12^

Rescue therapy was permitted at any time during the study if any of the following criteria were met in the study eye: A decrease of ≥10 letters in ETDRS BCVA from baseline (day 0); an increase in CST of ≥100 µm or 20%, whichever is lower, from baseline (day 0) based on the CST measurement at the clinical site; a ≥ 1.5 step increase from baseline (day 0) in the level of inflammation (e.g., anterior chamber cells or vitreous haze) or an increase from 3+ to 4+; and/or the investigator judged the uveitic complications in the study eye had not improved and the condition required additional treatment. The choice of rescue therapy was left to the discretion of the investigator.

### 2.2 Efficacy and safety endpoint assessments

Efficacy endpoints evaluated in this post hoc analysis included: Proportion of subjects with ≥15‐letter improvement from baseline in BCVA, mean change from baseline in BCVA letter score, mean change from baseline in CST and proportion of subjects with retinal thickness < 300 µm, all at 24‐weeks. Safety endpoints evaluated included mean change from baseline in IOP at 24‐weeks and incidences of IOP elevations ≥30 mm Hg, proportions of subjects requiring ≥1 additional IOP‐lowering medications and proportions of subjects requiring surgical intervention for IOP. All IOP endpoint assessments omitted IOP values measured immediately following the study treatment injection procedures to exclude procedure related, volume driven events. Safety endpoints also included the incidences of serious AEs (SAEs) and treatment‐emergent AEs (TEAEs) reported during the entire 24‐week study period.

### 2.3 Statistical analysis

In this post hoc analysis, endpoints were evaluated in the subset of CLS‐TA subjects from the PEACHTREE intent‐to‐treat (ITT) population (all randomised subjects) who did not require rescue therapy and the subset of subjects in the control group that received at least one rescue therapy at any time during the study up to Week 24. Descriptive statistics were used to summarise continuous variables, while counts and percentages were used to summarise categorical variables. The number of subjects who received rescue was summarised. Demographic and disease characteristics were evaluated across subsets in order to assess for clinically important imbalances.

The number and percentage of subjects with an improvement from baseline of 15 letters or more in BCVA and a CST of less than 300 μm were summarised. Differences between treatments were tested using a Cochran–Mantel–Haenszel chi‐square test stratified by country. For mean changes from baseline in BCVA and CST the estimate of the between‐treatment difference, 95% confidence interval and *p* value were calculated based on an analysis of variance (ANOVA) model with fixed effects for treatment group and country.

Endpoints based on the assessment of IOP were enumerated and included a clinically relevant IOP of ≥30 mm Hg. IOP lowering medications and IOP‐lowering surgeries were enumerated. The number and percentage of subjects experiencing TEAEs or SAEs during the study were summarised. All analyses were done on the ITT population as observed; no methods were used to impute values for missing data. No adjustments were made to the alpha level to account for multiple comparisons. Due to the post hoc nature of this analysis, and the fact that data from the two groups being compared do not reflect random samples, *p* values are nominal and are presented for descriptive purposes only. For the purpose of informing future studies, a *p* value ≤0.050 was considered statistically significant. All analyses and summaries were produced using SAS® version 9.4 (SAS Institute, Cary, NC) or higher for Windows.

## 3 RESULTS

### 3.1 Baseline subject characteristics

PEACHTREE randomised a total of 160 subjects (96, CLS‐TA; 64, control).^12^ Of these, 83/96 (86.5%) CLS‐TA subjects did not receive any rescue treatment and 46/64 (71.9%) control subjects received at least one rescue treatment during the study and qualified for inclusion in this post hoc analysis. Of the qualifying subjects, 79/83 (95.2%) CLS‐TA subjects and 46/46 (100%) control subjects completed the study. For the four subjects in the CLS‐TA group that were not rescued and did not complete the study: one was for non‐compliance, two withdrew consent and one subject was lost to follow‐up.

The two treatment groups were balanced with respect to age, race, gender, the mean duration of ME diagnosis in the study eye (69.2 weeks, CLS‐TA; 69.7 weeks, control), the anatomical location of uveitis and the onset type, duration and course of uveitis (Table 1 and Table 2). An imbalance in the time since uveitis diagnosis was noted with the unrescued CLS‐TA subjects having a longer mean duration (177.1 weeks) as compared to the rescued control subjects (120.0 weeks). Key ophthalmic assessments at baseline showed no significant differences between unrescued CLS‐TA subjects compared to rescued control subjects. Specifically, in the unrescued CLS‐TA subjects, mean baseline BCVA was 55.6 letters and in the rescued control subjects mean baseline BCVA was 53.8 letters (95% CI for difference: ‐2.9 to +7.0 letters, *p* = 0.414, ANOVA). In the unrescued CLS‐TA subjects, mean baseline CST was 480.6 μm and in the rescued control subjects mean baseline CST was 519.7 μm (95% CI for difference: ‐95.1 to +20.8 μm, *p* = 0.207). In the unrescued CLS‐TA subjects, mean IOP at baseline was 13.5 mm Hg and in the rescued control subjects mean baseline IOP was 13.3 mm Hg.

**TABLE 1:** Demographics summary in unrescued CLS‐TA subjects versus rescued control subjects

Demographic	Unrescued CLS‐TA (*N* = 83)	Rescued Control (*N* = 46)
Gender, *n* (%)		
Male	35 (42.2)	21 (45.7)
Female	48 (57.8)	25 (54.3)
Race, *n* (%)		
American Indian or Alaskan Native	0 (0.0)	0 (0.0)
Asian	41 (49.4)	18 (39.1)
Black or African‐American	8 (9.6)	8 (17.4)
Native Hawaiian or Other Pacific	0 (0.0)	0 (0.0)
Islander	33 (39.8)	20 (43.5)
White	1 (1.2)	0 (0.0)
Other		
Ethnicity, *n* (%)		
Hispanic or Latino	8 (9.6)	6 (13.0)
Not hispanic or Latino	75 (90.4)	40 (87.0)
Age, years		
*N*	83	46
Mean (SE)	49.7 (1.53)	50.0 (2.22)
SD	13.92	15.08
Median	50.0	51.0
Minimum, maximum	18, 78	22, 85

**TABLE 2:** Baseline disease characteristics in the study eye in unrescued CLS‐TA subjects versus rescued control subjects

Baseline Disease Characteristic	Unrescued CLS‐TA (*N* = 83)	Rescued control (*N* = 46)
Time since uveitis diagnosis, weeks		
*N*	83	46
Mean (SE)	177.1 (25.25)	120.0 (21.23)
SD	230.04	143.97
Median	73.0	69.5
Minimum, maximum	0, 1262	0, 648
Anatomic location of uveitis, *n* (%)		
Anterior	26 (31.3)	11 (23.9)
Intermediate	30 (36.1)	15 (32.6)
Posterior	20 (24.1)	9 (19.6)
Pan	22 (26.5)	18 (39.1)
Onset type, *n* (%)		
Sudden	19 (22.9)	10 (21.7)
Insidious	64 (77.1)	36 (78.3)
Duration, *n* (%)		
Limited (≤ 3 months)	16 (19.3)	8 (17.4)
Persistent (> 3 months)	67 (80.7)	38 (82.6)
Course of uveitis, *n* (%)		
Acute	5 (6.0)	5 (10.9)
Recurrent	28 (33.7)	14 (30.4)
Chronic	50 (60.2)	27 (58.7)
Time since macular oedema diagnosis, weeks		
*N*	83	46
Mean (SE)	69.2 (24.11)	69.7 (18.33)
SD	219.61	124.35
Median	7.0	10.0
Minimum, maximum	0, 1888	0, 651
Presence of, *n* (%)		
Macular oedema	83 (100)	46 (100)
Foveal reflex	13 (15.7)	7 (15.2)
Intraretinal fluid	74 (89.2)	40 (87.0)
Subretinal fluid	24 (28.9)	10 (21.7)
Fibrosis	2 (2.4)	2 (4.3)

### 3.2 Rescue therapy in control arm

Among the 46 rescued control subjects, the Kaplan–Meier median time to first rescue was 84 days; with one patient being rescued as early as 7 days, 20 being rescued within the first month and 32 being rescued prior to Week 12.^12^ First rescue most commonly consisted of corticosteroids administered topically (39.1%), intravitreally (30.4%), systemically (13.0%), or periocularly (10.9%). Topical non‐steroidal anti‐inflammatory drugs were administered for few (6.5%) first rescues. Many of the patients who were initially rescued with topical corticosteroids required additional, escalating therapy; ultimately, 63.0% of control subjects received corticosteroids intravitreally and 17.4% control subjects received corticosteroids periocularly, with the remaining receiving corticosteroids topically (15.2%) or systemically (4.3%). Overall, IVT or periocular corticosteroids were ultimately administered in 37/46 (80.4%) of rescued control subjects.

### 3.3 Efficacy

The percentage of subjects with a gain from baseline of ≥15 letters in BCVA was 51.9% (41/79) in the unrescued CLS‐TA group compared to 37.0% (17/46) in the rescued control group at Week 24 (95% CI for difference: ‐3.3% to +32.5%, *p* = 0.115).

At Week 24, unrescued CLS‐TA subjects who completed the study showed a mean improvement of 15.7 letters, compared to a 10.9 letter improvement in the rescued control subjects who completed the study (95% CI for difference: ‐0.5 to +9.6 letters, *p* = 0.080).

Analysis of CST showed a similar trend. Of the 77 unrescued CLS‐TA subjects with gradable images at Week 24, a total of 47 (61.0%) had a CST < 300 μm compared to 22/44 (50.0%) rescued control subjects (95% CI for difference: ‐7.7% to +29.2%, *p* = 0.322).

**FIGURE 1:**
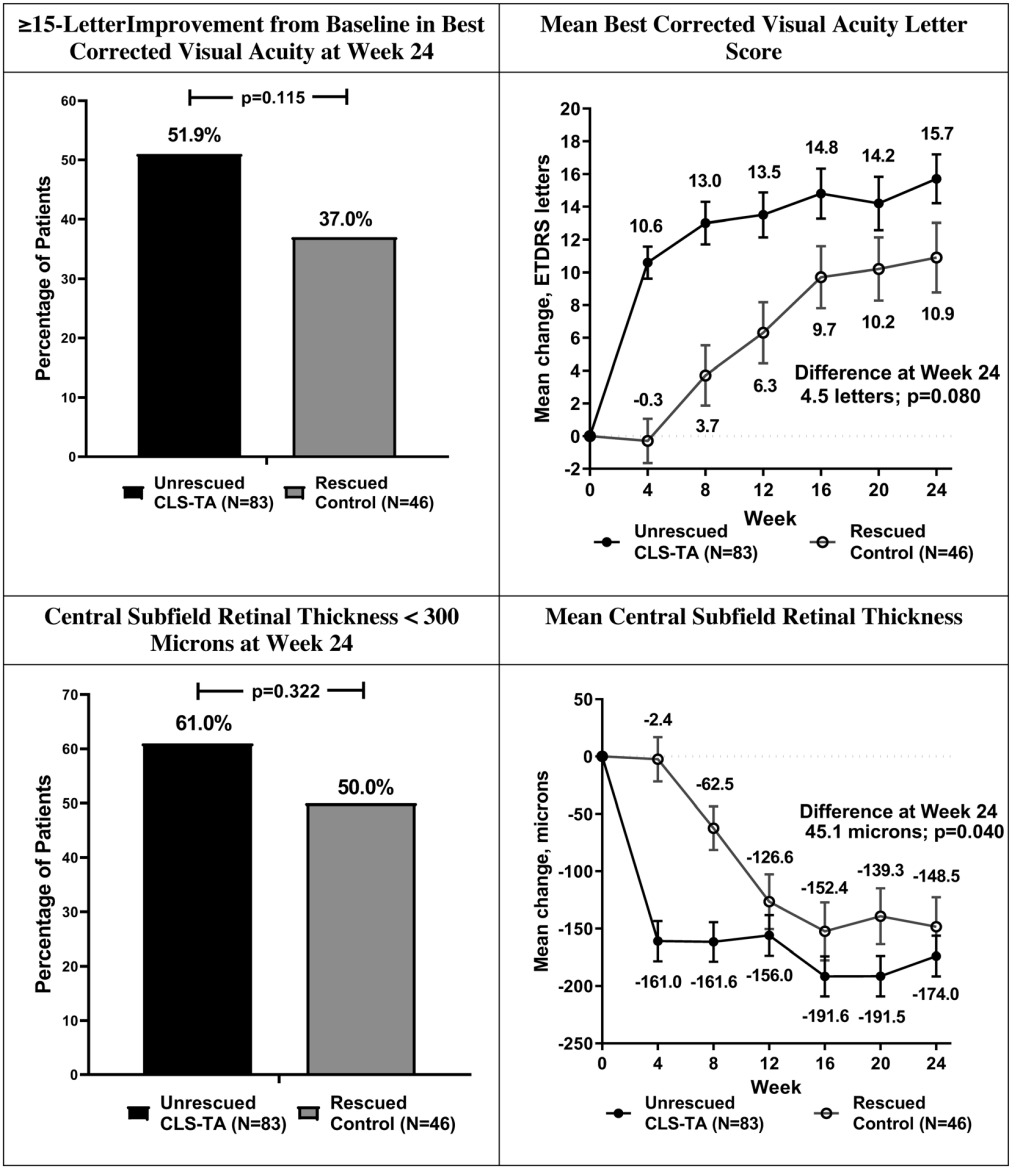
Efficacy summary: Visual acuity and central subfield thickness through Week 24 in unrescued corticosteroid formulation‐triamcinolone acetonide (CLS‐TA) subjects versus rescued control subjects. The difference and p‐value are derived from the analysis of variance model

At Week 24, the unrescued CLS‐TA subjects who completed the study with gradable images showed a 174.0 μm reduction, compared to a 148.5 μm reduction in the rescued control subjects (95% CI for difference: ‐88.2 to −2.0 μm, *p* = 0.040). (See Figure 1).

An additional analysis excluding patients receiving baseline systemic therapy was consistent with the above results; unrescued CLS‐TA patients (n = 58) trended toward better BCVA and CST outcomes than rescued control patients (n = 34).^15^

### 3.4 Safety

At Week 24, unrescued CLS‐TA subjects who completed the study showed a mean IOP of 15.2 mm Hg, representing an increase from baseline in IOP of 1.6 mm Hg. Rescued control subjects showed a mean IOP of 15.4 mm Hg at Week 24 reflecting a mean increase of 2.0 mm Hg from baseline.

**FIGURE 2:**
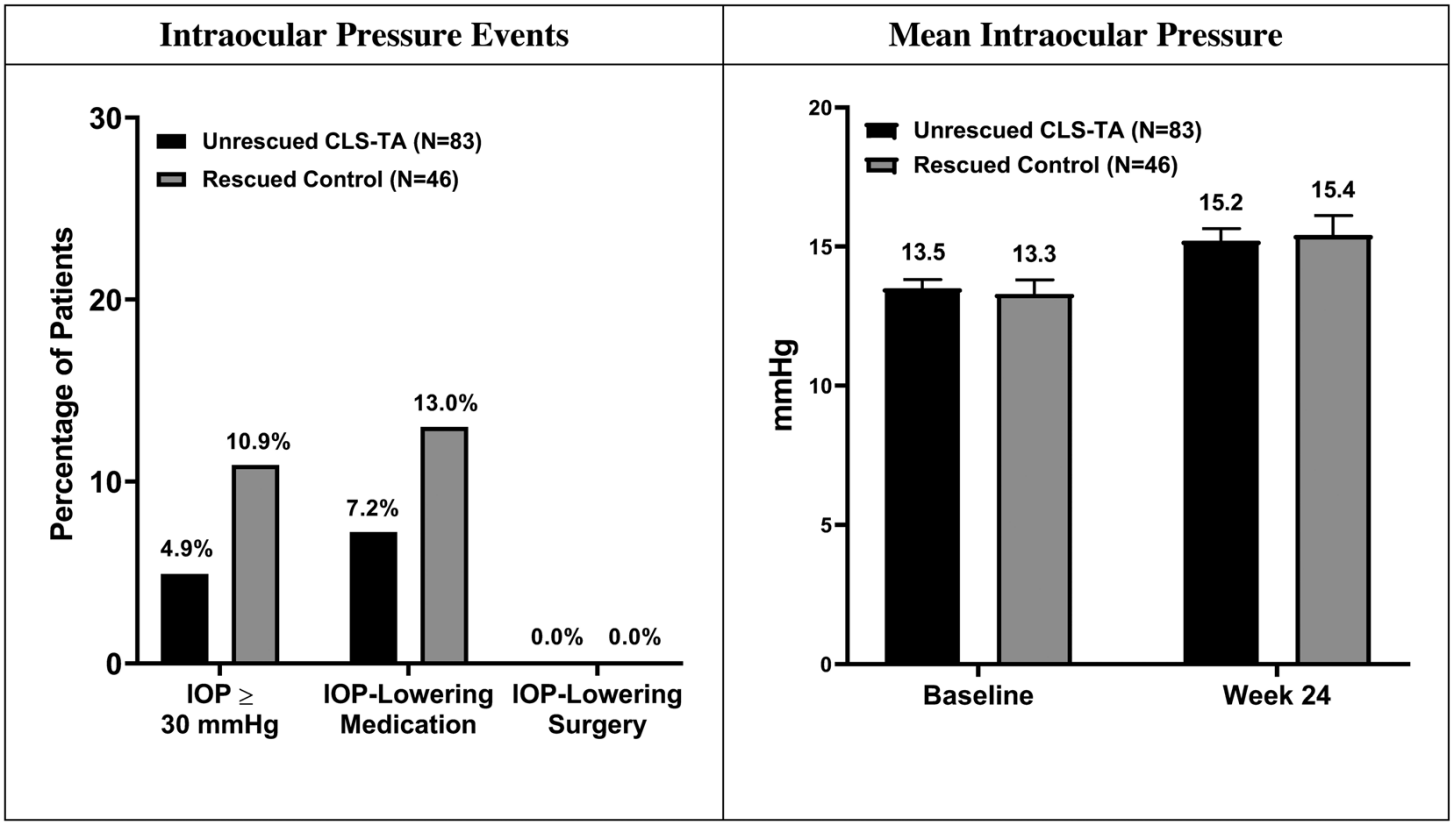
Intraocular pressure related events through Week 24 in unrescued corticosteroid formulation‐triamcinolone acetonide (CLS‐TA) subjects versus rescued control subjects

Unrescued CLS‐TA subjects tended to experience fewer IOP‐related events with lower incidents of IOP ≥30 mm Hg at any visit compared to control subjects who received rescue therapies (4.9% vs. 10.9%). No subject experienced an IOP over 37 mm Hg. The use of IOP lowering medications in subjects who only received CLS‐TA appeared lower than control subjects who received rescue therapy (7.2% vs. 13.0%) (See Figure 2). No subject underwent surgery to treat elevations in IOP.

The percentage of subjects with ≥1 TEAE in the study eye was 48.2% in the unrescued CLS‐TA subjects and 63.0% in the rescued control subjects. AEs pertaining to elevated IOP occurred in 10.8% of unrescued CLS‐TA subjects and in 21.7% of the rescued control subjects. Cataract incidence appeared lower in the unrescued CLS‐TA subjects than in the rescued control subjects (4.8% vs. 8.7%, respectively). (See Table 3).

**TABLE 3:** Ocular adverse events by rescue therapy and treatment group

Study eye, *n* (%)	No Rescue		Rescue	
	CLS‐TA (*N* = 83)	Control (*N* = 18)	CLS‐TA (*N* = 13)	Control (*N* = 46)
Total number of ocular adverse events	97	9	25	45
Number of patients with ≥1 ocular AE	40 (48.2)	8 (44.4)	9 (69.2)	29 (63.0)
Treatment‐related ocular AEs	23 (27.7)	2 (11.1)	6 (46.2)	6 (13.0)
Serious ocular AEs	0	0	1 (7.7)	0
Treatment‐related serious AEs	0	0	0	0
TEAEs leading to study drug discontinuation	2 (2.4)	0	3 (23.1)	5 (10.9)
Number of patients with ≥1 eye disorder	32 (38.6)	6 (33.3)	9 (69.2)	28 (60.9)

Adverse events				
Cataract^a^	4 (4.8)	0	3 (23.1)	4 (8.7)
Cystoid macular oedema	0	1 (5.6)	0	8 (17.4)
Eye pain^b^: Time of procedure	9 (10.8)	2 (11.1)	3 (23.1)	1 (2.2)
Eye pain: Any time post procedure	5 (6.0)	0	1 (7.7)	0
Elevated IOP^c^: Time of procedure	8 (9.6)	0	0	0
Elevated IOP^c^: Pertaining to corticosteroid^d^	9 (10.8)	0	2 (15.4)	10 (21.7)
Uveitis	0	0	2 (15.4)	7 (15.2)
Vitreous detachment	3 (3.6)	1 (5.6)	2 (15.4)	0

^a^ Cataract includes the preferred terms (a) cataract, (b) cataract subcapsular and (c) cataract nuclear.

^b^ “Eye pain” includes the preferred terms (a) eye pain, (b) injection site pain, (c) injection site discomfort.

^c^ “Elevated IOP” includes the preferred terms (a) IOP increased, (b) ocular hypertension and (c) glaucoma.

^d^ Includes all events of elevated IOP that did not occur on the day of the procedure.

One SAE was reported in the unrescued CLS‐TA group: Post‐traumatic lumbar compression fracture that was not considered related to treatment by the investigator and Sponsor and did not lead to discontinuation from the study.

## 4 DISCUSSION

PEACHTREE was a sham‐controlled study and was not designed to compare suprachoroidal administration of CLS‐TA to other ocular or systemic corticosteroid treatments for uveitic ME in a controlled fashion. However, based on pre‐defined criteria, rescue therapy could be administered to both subjects in the CLS‐TA group or sham‐controlled group, thus in effect creating a surrogate active comparator arm (i.e., control subjects receiving rescue). In this post hoc analysis, the primary objective then was to compare safety and efficacy results from the unrescued CLS‐TA subjects in PEACHTREE to that of rescued control subjects (surrogate active comparator arm). Rescue therapy in the control arm consisted primarily of IVT and/or periocular corticosteroids.

Results of this post hoc analysis are consistent with the results reported for PEACHTREE.^12^ Specifically, subjects who only received CLS‐TA showed consistent trends of greater visual gains, greater reductions in CST and lower rates of IOP events and cataracts relative to subjects in the control arm who received rescue therapy. Similar trends for ≥15 letters in BCVA, mean improvement in BCVA and CST were noted in patients not receiving systemic corticosteroid and /or immunomodulatory therapies at baseline. Although most between‐group differences were not statistically significant, likely due to the small sample size, the efficacy and IOP findings for CLS‐TA largely align with pre‐clinical ocular distribution studies. Namely, in these pre‐clinical studies, suprachoroidal injection of CLS‐TA yielded high levels of the corticosteroid in the retina, retinal pigmented epithelium and choroid, detectable for over 3 months, with limited exposure to the anterior segment, as compared to IVT injection suggesting that CLS‐TA administered suprachoroidally has the potential to target the posterior segment while decreasing corticosteroid related AEs such as elevated IOP and cataract.

There is limited data on the comparative effectiveness of therapies for uveitic ME. The PeriOcular and INTravitreal Corticosteroids for Uveitic Macular Edema Trial (POINT) study, an NIH‐funded prospective clinical trial, compared three commonly administered local corticosteroids for uveitic ME. At 6 months, IVT triamcinolone acetonide and IVT dexamethasone implant outperformed periocular corticosteroid, both achieving approximately nine letters of improvement compared to approximately four letters, respectively.^14^ Although cross‐trial comparisons have limitations, the current analysis showed that unrescued CLS‐TA subjects experienced a mean 6‐month improvement of 15.7 letters, compared to a 10.9 letter improvement in the rescued control subjects. With respect to safety, the POINT study demonstrated that approximately 24%–33% of subject in each arm required IOP‐lowering medication. In the current analysis, 7.2% unrescued CLS‐TA subjects required IOP‐lowering medications, compared to 13.0% in the rescued control subjects.

Limitations of this study include its sample size and post hoc design, which cannot replace a prospective comparative effectiveness trial. One source of potential bias, likely related to the post hoc nature of this study, is that the two groups are not necessarily homogeneous, resulting in potential differences in disease characteristics. In addition, while there were pre‐defined criteria for rescue therapy administration, the choice of rescue therapy and route of administration were at the investigators' discretion. Thus, rescue medication use in the control group comprised a heterogenous mixture of medications/routes as compared to a single comparator agent/administration route, yet provided a real‐world snapshot of current practice. Although all rescued control subjects ultimately received corticosteroids, including IVT or periocular corticosteroids in 80.4% of rescued control subjects, CLS‐TA subjects had longer corticosteroid exposure compared to rescued control subjects, possibly biasing efficacy measures in favour of the unrescued CLS‐TA group and safety measures in favour of rescued control group. In a recently published paper, longitudinal modeling for CLS‐TA treated eyes showed that CST reached 90% of maximal improvement before the analogous 90% maximal response in BCVA was observed.^15^ Despite these limitations, the efficacy and safety results of this post hoc study appear consistent with the findings of PEACHTREE and are of interest to clinicians who treat subjects uveitic ME. Further, randomised comparative effectiveness studies are warranted.

## CONFLICT OF INTEREST

Dr. Singer reports receiving consulting fees from Allergan, Aerie, Genentech, Regeneron, Novartis and Eyepoint as well as grant support from Optos, Appelis, Kodiak, Unity, Astellis, Ionis, Clearside, Allergan, Aerie, Genentech, Regeneron, Novartis and Eyepoint. Dr. Merrill reports receiving consulting fees from Santen, Gilead and Eyepoint as well as grant support from Clearside. Dr. Yeh reports receivning consulting fees from Clearside, Adverum and Santen. Dr. Hall, Dr. Ciulla and Mr. Kapik are employees of Clearside and hold stock in the company.

## Data Availability

The efficacy results presented in this manuscript were included in the primary PEACHTREE publication by Yeh. Data from this manuscript were presented at the Retina Society Annual Meeting 2020, the American Society of Retina Specialists Annual Meeting 2020, the Macula Society Annual Meeting 2020 and the Association for Research in Vision and Ophthalmology Annual Meeting 2020.
